# Care coordination in pediatrics: Experience of the outpatient clinic for Children with Special Health Care Needs (CSHCN)

**DOI:** 10.1016/j.clinsp.2022.100049

**Published:** 2022-05-22

**Authors:** Ana Paula Scoleze Ferrer, Daleth Rodrigues Scaramuzzi, Maria Lúcia M. Bourroul, Sandra M.C. Zuccolotto, Sandra J.F.E. Grisi

**Affiliations:** Departamento de Pediatria, Hospital das Clínicas, Faculdade de Medicina, Universidade de São Paulo (HCFMUSP), São Paulo, SP, Brazil

Children with conditions that until a few years ago were incompatible with life currently survive due to technological advances, changing the epidemiological profile and leading to the need to reassess the healthcare.^1^ These patients often present with chronic comorbidities and sequelae, which require healthcare of different quality compared to the general pediatric population, is defined as “Children with Special Health Care Needs” (CSHCN) ‒ patients with a higher risk of chronic physical problems and developmental, behavioral, or emotional alterations and require healthcare in greater density or of different quality compared to the general pediatric population.^2^

CSHCN presents quite heterogeneous conditions regarding clinical manifestations, severity, and functional limitations and, consequently, varied health care requirements, which interferes with the organization of care and adoption of programs or specific health policies.^3^ In this way, there is a subgroup of CSHCN who need more intensive care, defining them as carriers of “complex chronic conditions” (CCC).^2^

CCC involves the combination of four characteristics: (1) Presence of one or more severe chronic clinical conditions and/or association with higher morbidity and mortality rates; (2) Functional impairment and mental and/or physical limitations, often leading to compromised autonomy and/or need of assistive technology (gastrostomy, tracheostomy) to ensure survival; (3) Different care needs compared to the general population, such as polypharmacy, specialized therapies, and special diets, besides specific educational needs; and (4) Greater use of healthcare services, with a higher number of medical appointments, multiple professionals from different areas involved in the care, and more frequent and/or prolonged hospitalizations.^2^

In the United States, there is an estimated prevalence of 15–20% of CSHCN, responsible for the high healthcare costs, even though complex cases are rare.^2^,^3^ There is much evidence of deficiency of the healthcare system in meeting the demands of these patients.^1^ The fragmentation of care results in multiple consultations and therapies making it difficult for the family's understanding and adherence to the proposed treatments. Therefore, it is necessary to seek assistance models that can improve the management of care for children and support caregivers. To minimize the undesirable impacts, the organization of care based on care coordination has been proposed.^3^, ^4^, ^5^

Care coordination is a strategy of approach, with emphasis on inter-organizational relationships,^4^ and has been described as the best way to respond to the different demands of families of CCC, resulting in improved caregiver satisfaction, health conditions, and decreased hospitalization and better use of medical services.^3^, ^4^, ^5^

This care model has been recommended by the American Academy of Pediatrics for care to CSHCN under the denomination of Medical Home, which encompasses a broad concept of an “accessible, comprehensive, longitudinal, coordinated, humanized, culturally adapted, and family-centered” approach. According to these characteristics, care for these patients can be provided at any level of care. However, it should be ideally provided in primary care, usually by a general pediatrician with the joint support of other specialists and a multidisciplinary team, with care coordination being the key element.^6^,^7^

The ICR-HCFMUSP outpatient clinic for CSHCN and CCC was established in 2016 with the following objectives: (1) To provide coordinated outpatient care to patients with CCC, aiming to integrate the multispecialty assistance that these patients require; (2) To facilitate dehospitalization and support families in understanding the treatment; (3) To train pediatric resident physicians with a view to disseminate and implement the model in other locations; and (4) To contribute to the production of knowledge in this field.

Since 2016, 1006 patients referred from the several specialties clinics of the HCFMUSP were examined, totaling 8,028 appointments. The main characteristic of the patients is the multiplicity of health problems. Most of them present three or more diagnoses need polypharmacy, and approximately one-third are technology-dependent and/or need assistive technology, like gastrostomy, tracheostomy, supplemental oxygen, and others.

Care is provided by the pediatric resident physician under the supervision of a general pediatrician who identifies all morbid conditions and respective therapeutic proposals guided by specialists, addresses the needs of the patient and the family, establishes priorities, and shares decisions. Through dialogue with specialists and a multi-professional team, a therapeutic care plan is created, including clinical issues, aspects related to the promotion of quality of life, autonomy, and educational and social insertion of the child and adolescent.

This is the profile of the service that has been characterized as a service of “complex pediatric care”. However, the proposal of an outpatient clinic for the follow-up of children with CCC encounters some barriers.

The first dare is to find professionals in primary care who feel able to follow these patients, especially in aspects related to the integration of inpatient and outpatient care coordination.^8^ Professional qualifications can favor the healthcare system organization, having the general pediatrician responsible for coordinating care in the longitudinal follow-up and promoting the interface with the necessary specialties, including palliative care.^7^ Another necessary integration is with an adult practitioner, by means of the organization of outpatient clinics for the transition of care for those patients who reach adulthood, allowing adequate continuity of care.^9^ An agile and adequate referral and the counter-referral system are another difficult to overcome.

The dehospitalization of these patients, especially those technology-dependent, and the prevention of injuries depend on the qualification of the multi-professional team.^10^ In our experience, the authors observed limited access to multi-professional care and equipment. Optimization of resources and greater access to rehabilitation services is fundamental for reducing the need for emergency care services and hospitalizations, with positive impacts on the quality of life of patients and their families.

In addition to access and technical training to assist these patients, it is necessary to overcome difficulties of integration between the health sector and others. Care coordination presupposes communication with the area of education, social assistance and non-governmental organizations, and family groups.^6^

The difficulty in offering the necessary attention to patients with CCC has been the subject of increasing attention and should be the focus of discussions on the organization of services. The experience described allows for reflection and points to care coordination by a general practitioner as a strategy to improve health outcomes, both from an individual and family perspective and on the healthcare system. However, several barriers need to be overcome.

## Conflicts of Interest

The authors declare no conflicts of interest.
